# Ultrasound-Guided Percutaneous Needle Electrolysis in Dancers with Chronic Soleus Injury: A Randomized Clinical Trial

**DOI:** 10.1155/2020/4156258

**Published:** 2020-08-27

**Authors:** B. De-la-Cruz-Torres, I. Barrera-García-Martín, F. Valera-Garrido, F. Minaya-Muñoz, C. Romero-Morales

**Affiliations:** ^1^Department of Physiotherapy, University of Seville, Avicena Street 41009, Seville, Spain; ^2^MVClinic Institute, Madrid, Spain; ^3^CEU San Pablo University, Madrid, Spain; ^4^Getafe FC., Madrid, Spain; ^5^Faculty of Sport Sciences, Universidad Europea de Madrid, Villaviciosa de Odón, Madrid, Spain

## Abstract

Damage to intramuscular tendons is very common in sports injuries, specifically in soleus muscle injuries. This study sought to compare the effects of applying ultrasound- (US-) guided percutaneous needle electrolysis (PNE) in combination with an eccentric exercise program on pain and functionality in dancers with chronic soleus injury, located in the central tendon. Thirty dancers with injured central tendon of the soleus muscle were randomly allocated to a PNE group (*n* = 10), an eccentric exercise group (*n* = 10), or a combined group (*n* = 10). Pain, ankle dorsiflexion range of motion (DROM), endurance, the heel raise test, the DFOS questionnaire, and the minimal clinically important difference (MCID) were analyzed at baseline and after treatment (four weeks). Over half (52%) of the dancers had a chronic soleus muscle injury. Variables for pain, DROM, the heel rise test, ADL, technique, DFOS total, and DFOS-subjective variables showed significant differences (*P* < 0.05) in pretreatment and posttreatment in all groups, whereas no significant differences were observed between intervention groups. However, the combined group showed a higher percentage of changes compared to the other groups, and these dancers had greater perceived changes (MCID = 4.70 ± 1.42). The conclusion of the study was that dancers with chronic soleus injury, located in the central tendon, treated with a combination of US-guided PNE and an eccentric exercise program displayed improved outcomes compared to the application of PNE therapy or eccentric exercise alone. The US-guided PNE, combined with an eccentric exercise program, is a useful therapeutic tool for the treatment of chronic soleus injury, located in the central tendon. The trial is registered with NCT04042012.

## 1. Introduction

The soleus muscle is located in the posterior region of the lower leg and is composed primarily of slow twitch (type I) fibers. Its main function is to perform low-speed activity, such as walking and postural control [1]. The soleus muscle has both lateral and medial intramuscular aponeuroses [2] and an intramuscular tendon (IMT), located in the central part of the muscle, which contributes to the formation of the Achilles tendon [3]. The IMT is considered as being a central supporting strut to which the muscle fibers attach.

Soleus muscle injuries have traditionally been considered difficult to diagnose [4]. This is because injuries involving the soleus muscle have a varied topography, depending on the affected musculotendinous union. Balius et al. [5] suggested five potential sites in the soleus muscle where lesions may be located: myofascial sites (anterior strains and posterior strains) and musculotendinous junction sites (proximal lateral strains, proximal medial strains, and distal central tendon strains).

Damage to the IMTs constitutes a very common sports injury, specifically in the case of soleus muscle injuries. The IMTs are characterized by being considerably stiffer (strains of 2%–2.6%); intramuscular tendon ruptures result in bleeding and a response of inflammation, proliferation, and maturation, resulting in the formation of hypertrophic intramuscular tendon scar tissue; furthermore, athletes present symptoms such as progressive tightness [6]. These muscle injuries are often misdiagnosed and have an insidious evolution, and athletes often have a high risk of reinjury. This might be the result of a lack of specific treatment protocols for these muscle injuries [7, 8].

Currently, a technique known as percutaneous needle electrolysis (PNE) is also being used. Ultrasound- (US-) guided PNE is a novel, minimally invasive approach that involves the application of a galvanic current through an acupuncture needle. This technique stimulates a local inflammatory response and influences in the vascularization of the injured area [9, 10]. The needle is placed in the injured soft tissue using ultrasound. This procedure has mainly been applied to treat tendinopathies [11–16], plantar fasciosis [17], whiplash syndrome [18], subacromial pain syndrome [19, 20], and temporomandibular myofascial pain [21]. However, little is known regarding its effectiveness in muscle injuries. Abat et al. [9] and Santafé et al. [22] have studied the effect of PNE in muscle injuries among animal models; however, to date, no clinical trials have studied the effects of PNE in humans. To the best of our knowledge, only case series have been carried out, with good clinical results. Jiménez-Rubio et al. [23] applied US-guided PNE on acute muscle injuries in two cases and suggested that a combined treatment using PNE and a functional exercise program may reduce the time for return to competition after a hamstring injury.

The aim of this study was to compare the effects of adding US-guided PNE to an eccentric exercise program on pain, range of motion of the ankle dorsiflexion, and functionality in dancers with chronic soleus muscle injury.

## 2. Materials and Methods

### 2.1. Design

The present study was a randomized clinical trial, reported using the Consolidated Standards of Reporting Trials (CONSORT) guidelines.

### 2.2. Participants

**Figure 1:**
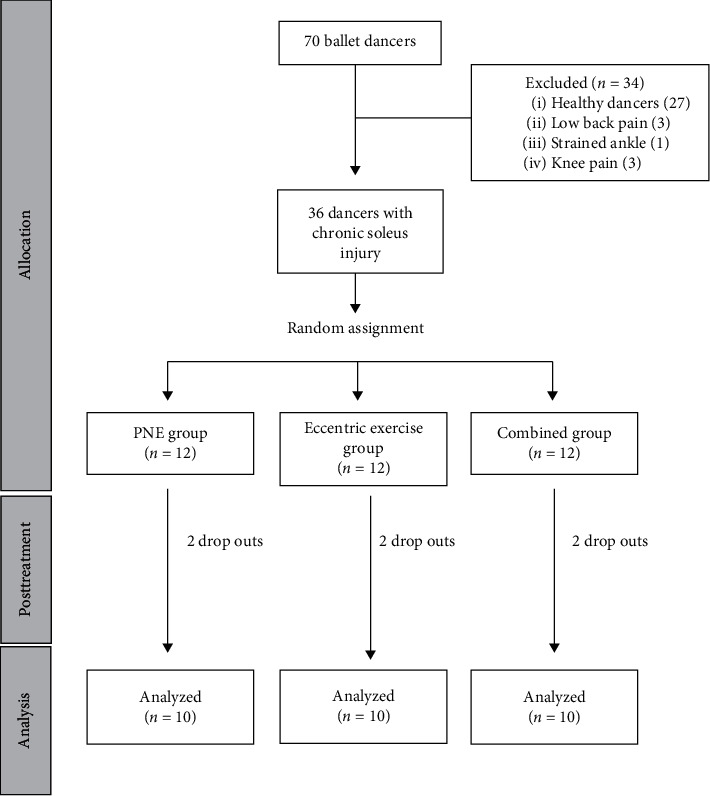
CONSORT flow diagram of dancer recruitment and retention.

**Figure 2:**
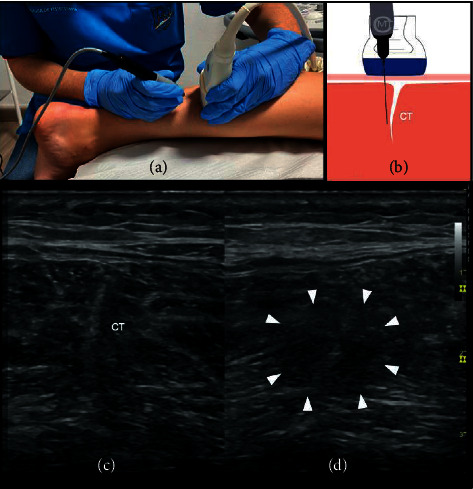
PNE intervention in soleus muscle injury. (a) Ultrasound-guided invasive approach of the soleus muscle; (b) ultrasound-guided percutaneous needle electrolysis procedure; (c) normal ultrasound image of the soleus muscle (transverse axis); and (d) ultrasound image of the injured central tendon of the soleus muscle (transverse axis). Ultrasound imaging shows muscle fiber disorganization at the level of tear (arrowhead). CT: central tendon.

The target sample was classical dancers from the local dance conservatory and official dance company (70 dancers in total). After applying the inclusion/exclusion criteria, a total sample of 36 full-time dancers (52%) with chronic soleus muscle injury, located in the central tendon, was recruited for this study. Over the course of the study, 6 dancers dropped out. Ultimately, 30 ballet dancers (mean age 21.03 ± 2.88 years old, range 16–26) completed the study. The flowchart of the recruitment process is given in Figure 1. The inclusion criteria were (a) ballet dancers; (b) with a minimum of five years dance training; (c) training at least four hours, five days a week; and (d) having a chronic soleus muscle injury. The exclusion criteria consisted of (a) any pathology in the lower back and lower limbs; (b) commonly accepted contraindications to PNE [24]; (c) any contraindications to needling *per sé* [24]; and epilepsy. Specifically, a chronic soleus muscle injury, located in the central tendon, was considered by the authors, if this pathology met the following characteristics: the presence of symptoms for at least six months; a self-rated pain intensity score greater than or equal to 4 cm during dance activities, measured using the visual analogue scale (VAS); and a progressive tightness during dance activities. Prior to the physical examination, a clinical history was taken. The presence of a chronic central intramuscular tendon injury of the soleus muscle was assessed based on a clinical examination and clinical history of the dancers by an expert therapist and confirmed using ultrasound examination performed by an experienced clinician. Specifically, the ultrasound image showed a muscle fiber structure alteration at the level of central tendon of the soleus characterized by a diffuse hypoechoic or hyperechoic area, with disorganization and disruption of the central tendon line (Figure 2).

### 2.3. Ethical Considerations

The local ethics committee approved the study, which complied with all the principles set out in the Declaration of Helsinki. All subjects signed written informed consent to participate in this study. Before participation, the dancers and parents/guardians were fully informed of the protocol, and as the dancers were under the age of 18, written informed consent was obtained from their parents/guardians. This study was registered in the clinical trials database (clinicaltrials.gov), with NCT04042012. The dancers were recruited from dance companies and schools. The recruitment period was from August 1 to December 31, 2019.

### 2.4. Interventions

Participants were divided randomly into three groups, each comprising 10 participants: an eccentric exercise group, a US-guided PNE group, and a combined group, receiving both interventions.

#### 2.4.1. Eccentric Exercise Group

Participants in this group performed eccentric exercises of the soleus muscle. Each dancer required a small step to perform this exercise. Participants were instructed to perform eccentric soleus muscle exercises below the surface of the step, one daily, 4 days/week for four weeks. From a vertical position of the body and standing with all the body weight on the rear foot, with the knee flexed and the ankle joint in dorsiflexion, the soleus muscle was loaded eccentrically by causing the dancer to lower the heel under the rear foot. These exercises are based on the stretching-shortening cycle, using their own bodyweight. The eccentric exercises involve three sets of 15 repetitions [25], by the end of dance classes (in the morning).

#### 2.4.2. US-Guided PNE

Dancers in this group received two sessions of US-guided PNE therapy (one session per week, in the afternoon). The number of PNE applications in the different studies is varied [11–23]. In the case of chronic muscle injury, only two interventions were performed due to the clinical experience of the authors [24]. Specifically, the procedure was applied using a specific device (Physio Invasiva®, PRIM, Madrid, Spain), which produces a continuous galvanic current through the cathode (modified electrosurgical scalpel with the needle) while the patient holds the anode (handheld electrode). An US machine (S7, GE Healthcare, Wisconsin, USA) with a GE ML6-15 linear probe was used. During PNE, the dancers were placed in the prone position with their feet hanging off the table. Isopropyl alcohol and chlorhexidine (Lainco® 2%) were used to prepare the skin. Subsequently, an acupuncture needle measuring 0.30 mm × 40 mm (Physio Invasiva® needles, PRIM Physio, Spain) was inserted using a short axis approach, perpendicular to the surface of the skin (80°), until the damaged area was reached (Figure 2). The PNE technique was performed using an intensity of 2.5 mA, during 3 seconds, 3 times (2.5 : 3:3), according to the protocol by Valera-Garrido and Minaya-Muñoz [24]. A physiotherapist with 10 years' experience in ultrasound evaluation and in invasive therapy administered the PNE intervention.

#### 2.4.3. Combined Group

Dancers in this group received US-guided PNE therapy, and they performed eccentric exercises, in the same manner as performed by the US-guided PNE group and eccentric exercise group, respectively.

### 2.5. Clinical Measurements

Demographic data were obtained including gender, age, weight, height, body mass index (BMI), and pathological side.

Severity of average pain in the soleus muscle at palpation was evaluated using the numeric rating scale (NRS) (0, no pain; and 10 mm, worst pain).

Ankle dorsiflexion range of motion (DROM) was measured using the weight-bearing lunge test (WBLT), according to the protocol by Vicenzino et al. [26]. The dancers' position was as follows: the injured limb was positioned forward, and the heel firmly planted on the ground while they flexed the knee to the wall. The noninjured limb was placed behind the test foot. The maximum DROM was measured in cm and defined as the distance from the wall to the great toe, and based on the furthest distance, the foot was able to be positioned without the heel raising off the floor while the knee was able to touch the wall. The final scores were collected based on the mean of three repeated values for each test. A restricted movement was considered a score less than 9-10 cm [27]. The intraclass correlation (ICC) was used to determine the reliability of the measurements, and it was established on the 10 volunteered subjects as being sufficient for clinical measurement (ICC = 0.93).

Endurance test [28]. Dancers balanced in unilateral stance heel raise (demi-pointe position) with eyes open, until the point of fatigue. The test was finished when the dancer's foot completely touched the floor.

Unilateral heel raises the fatigue test [28]. This test was performed on the floor, and heel raises were performed to a metronome tempo of 30 beats/min. The number of heel raises completed until fatigue was reported. Fatigue was considered as the failure to maintain heel height or the tempo for at least three consecutive raises. Light touch on a wall was authorized to assist with balance.

The Dance Functional Outcome Survey (DFOS) [29] is a self-administered questionnaire to assess healthy state and symptom severity in injured dancers. The DFOS is a 14-item lower extremity and low back Likert-scale questionnaire. The instrument assesses a dancer's ability to accomplish activities of daily living (ADL, 40 points) and dance-specific movements (technique, 50 points) [29]. Total points are the sum of ADL and technical scores, with 90 representing full function without limitations. The last question of the DFOS requires the dancers to grade their dancing performance from 0 to 100, with 0 being the worst and 100 being the best.

The minimal clinically important difference (MCID) is the smallest change in a treatment outcome that a patient would identify as being important and which indicates a change in the patient's management. To calculate the MCID score at the dancers' level, the patients' satisfaction with the change was used [30–32]. At the end of the study, the dancers were also asked to provide a global rating of perceived changes in their affected limb by comparing “how well is your leg doing?” (–7, much worse; 0, without changes; and 7, much better).

All variables were measured at baseline and at the end of treatment (at 4 weeks).

### 2.6. Data Analysis

SPSS 23.0 software (IBM SPSS Statistics; NY: IBM) was used for data analysis. First, the Shapiro–Wilks test was employed for the normality assumption. Second, the analysis of variance (ANOVA) was used, considering the homogeneity of the sample. Third, a two-way ANOVA for repeated measures was developed to assess the effects of intrasubject (pre- and posttreatment groups) and intersubject (treatment groups) values on the dependent variables. In addition, Bonferroni´s correction post hoc analyses were carried out. The level of significance was set at *P* < 0.05 with an *α* error of 0.05 (95% confidence interval) and a desired power of 80% (*β* error of 0.2).

## 3. Results

Over half (52%) of the dancers had a chronic soleus muscle injury, located in the central tendon. Therefore, a total of 30 participants were treated in this study. With the numbers available, the sociodemographic data did not show significant differences between groups (Table 1). As displayed in Table 2, the NRS values showed significant differences (*P* < 0.05) between pretreatment and posttreatment and between intervention groups, revealing a decrease in values in favor of the PNE group. The NRS, DROM, heel rise test, ADL, technique, DFOS total, and DFOS-subjective variables showed significant differences (*P* < 0.05) in all groups between pretreatment and posttreatment; however, no significant differences were observed between intervention groups. Table 2 also reveals the differences (Δ, in %) between pretreatment and posttreatment in each group.

The MCID was 4.60 ± 1.51 for the PNE group, 2.70 ± 2.31 for the eccentric exercise group, and 4.70 ± 1.42 for the combined group.

Values are mean ± SD unless otherwise indicated. ^†^Significant differences (*P* < 0.05) between PNE and the eccentric group. ADL, activities of daily living; DFOS, Dance Functional Outcome Survey; DROM, dorsiflexion range of motion; NRS, numeric rating scale; ROM, range of motion.

## 4. Discussion

The main finding of this study was that the combination of US-guided PNE and an eccentric exercise program led to better outcomes after treatment compared to the sole application of exercises or PNE therapy in dancers with chronic soleus injury (Table 2). The results showed that all groups exhibited significant improvements from baseline, regarding pain, DROM, the heel rise test, and dance performance. However, the combined group showed a higher percentage of changes compared to the other groups (Table 2). We believe that there was no difference between the groups due to two reasons: (a) concerning pain attitudes and management, dancers display an increased level of tolerance for pain and effort [33]. Indeed, during this study, the dancers continued with classes despite experiencing pain. Pain was thus accepted as being a necessity; and (b) typically, dancers usually spend part of their classes performing the technical movement in *relevé*. This movement implies a shortened position of the soleus muscle, and therefore, it is not a movement that can aggravate the symptomatology. These results confirmed our initial hypothesis that PNE therapy may provide a beneficial effect on chronic soleus muscle injury.

In 2009, Jiménez-Rubio et al. [23] applied US-guided PNE on acute muscle injuries in two cases, and they suggested that a combined treatment using PNE and a functional exercise program may reduce the time for return to competition after hamstring injury. In fact, Jiménez-Rubio et al. [34] developed and validated a functional on-field program for the rehabilitation and readaptation of soccer players after a hamstring strain injury, which includes the application of PNE. To the best of our knowledge, this is the first trial study to apply PNE treatment to the soleus muscle.

A soleus muscle injury may be underestimated and misinterpreted as not being clinically important, due to its chronic clinical characteristics and the fact that, in many cases, this pathology does not require athletes to stop playing sports [6]. The diagnosis of these injuries is often delayed because clinicians have suggested that ultrasound is not a sensitive technique for detecting soleus tears compared with magnetic resonance imaging [4], although the sensitivity is enhanced by a thorough, anatomically based ultrasound examination [35]. In addition, Pedret et al. [36] suggested that the injuries located in the central tendon of the soleus muscle have a longer readaptation than injuries in other locations [37–40]. Therefore, it is necessary to analyze the effect of different treatments protocols to try to reduce recovery times. The relevance of this finding to clinical practice lies in the application of a combined treatment (eccentric exercise + PNE) to achieve greater therapeutic benefits. These results are similar to other studies that reached the same conclusion. Moreno et al. [41] demonstrated that the combination of PNE and active physical therapy may cause a greater and more rapid reduction of pain. De Miguel Valtierra et al. [19] and Arias-Burias et al. [20] only found differences for shoulder pain when they applied US-guided PNE combined with manual therapy or exercise in patients with subacromial pain syndrome. Also, Abat et al. [11, 12] showed good clinical and functional in patellar tendinopathy when they applied US-guided PNE combined with an eccentric exercise program. Rodríguez-Huguet et al. [15] reported that degenerative structural changes (a symptom of chronic lateral epicondylitis) were reduced after eccentric and stretching exercise associated with US-guided PNE. The authors of this study suggest that the inclusion of PNE in physiotherapy treatments not only has effects on pain but also on performance in dancers. In addition, another strength of the present study was that the dancers' level of satisfaction (MCDI) regarding the application of the invasive technique was higher in the groups where, specifically, PNE was applied (MCID = 4.70 ± 1.42 for the combined group, MCID = 4.60 ± 1.51 for the PNE group, and MCDI = 2.70 ± 2.31 for the eccentric exercise group).

This study has several limitations. First, US-guided PNE therapy was only applied with eccentric exercise; however, clinical therapists usually treat their patients using different techniques. Future studies should examine the effectiveness of several methods including PNE therapy in combination with other accepted techniques. Second, dancers perform an extensive amount of technical movement in *relevé* (plantar flexed ankle) where the soleus muscle is tightened (concentric contraction), and the dancer has more difficulty feeling pain in the muscle. It would be interesting to study the chronic soleus muscle injury in other sports, such as runners, where this muscle performs shortening-stretching cycles and may further limit the performance of the athletes. Third, the participants did not stop dancing. If the authors could have controlled the training loads, perhaps, the results would have been different. Fourth, there was no evaluation of a follow-up period after the treatment. Authors are interested in knowing the long-term effects of this technique in future studies. And, fifth, the convenience sample was recruited for this study, so these findings should be interpreted with caution. Despite these limitations, the current findings may provide relevant preliminary data that can help in future interventions with this patient population.

## 5. Conclusions

In conclusion, the application of the US-guided PNE technique combined with eccentric exercise produces a higher percentage of changes for pain, DROM, the endurance test, the heel rise test, and DFOS questionnaire scores compared with the application of US-guided PNE technique and eccentric exercise alone in patients with chronic soleus injury, located in the central tendon. US-guided PNE technique should be applied in the treatment of chronic muscle injuries, and future studies should increase the knowledge about its clinical applications.

## Figures and Tables

**Table 1:** Sociodemographic data of the sample.

Data	PNE group (*n* = 10)	Eccentric exercise group (*n* = 10)	Combined group (*n* = 10)	*P* value
Age, years	20.40 ± 2.63	21.30 ± 2.71	21.40 ± 2.71	0.709
Weight, kg	55.50 ± 7.07	56.00 ± 4.24	57.80 ± 8.25	0.727
Height, m	1.65 ± 6.71	1.65 ± 5.82	1.63 ± 5.69	0.742
BMI, kg/m^2^	20.40 ± 2.41	20.38 ± 1.36	21.48 ± 2.23	0.401
Practice time, hours	29.9 ± 6.59	32.30 ± 5.33	30.00 ± 6.66	0.633
Dance years, years	14.2 ± 3.39	12.6 ± 4.67	14.90 ± 3.78	0.428
Gender (F/M)	9/1	9/1	9/1	—
Pathological side (R/L)	3/7	2/8	2/8	—

BMI, body mass index; PNE, percutaneous needle electrolysis.

**Table 2:** NRS, endurance test, DROM, the heel raise test, activities of daily living (ADL), dance-specific movements (technique), DFOS total, and DFOS-subjective intrasubject effects.

Measure	PNE group	% Δ	Eccentric exercise group	% Δ	Combined group	% Δ	Intrasubject effects	
							Time value F (df); *P* (eta^2^)	Treatment *X,* time F (df); *P* (eta^2^)
*NRS* ^†^							*F* (1, 2) = 66.253; *P*=0.001 (0.710)	*F* (1,2) = 4.197; *P*=0.026 (0.237)
Baseline	8.2 ± 1.0	−58.75	7.8 ± 0.7	−24.07	7.9 ± 1.3	−53.59		
Posttest	3.2 ± 2.4		5.9 ± 2.3		3.6 ± 1.4				
*Endurance test*							*F* (1,2) = 0.876; *P*=0.358 (0.031)	*F* (1,2) = 0.776; *P*=0.470 (0.054)
Baseline	14.2 ± 10.0	4.15	12.0 ± 7.5	43.21	8.9 ± 4.6	22.73		
Posttest	13.1 ± 8.6		14.5 ± 6.8		11.4 ± 10.0				
*DROM*							*F* (1, 2) = 25.815; *P*=0.001 (0.489)	*F* (1,2) = 2.444; *P*=0.106 (0.153)
Baseline	8.7 ± 1.55	13.34	7.4 ± 3.0	28.19	7.0 ± 2.8	83.26		
Posttest	9.8 ± 1.27		8.4 ± 2.8		9.4 ± 1.2				
*Heel rise test*							*F* (1, 2) = 14.143; *P*=0.001 (0.344)	*F* (1,2) = 0.537; *P*=0.590 (0.038)
Baseline	19.2 ± 6.3	18.21	15.3 ± 5.4	27.51	16.9 ± 5.4	33.51		
Posttest	21.9 ± 5.72		18.3 ± 7.27		21.1 ± 4.38				
*ADL*							*F* (1, 2) = 15.077; *P*=0.001 (0.358)	*F* (1,2) = 0.224; *P*=0.801 (0.016)
Baseline	35.7 ± 2.7	8.86	35.0 ± 6.2	9.86	32.7 ± 5.9	7.10		
Posttest	38.7 ± 1.7		37.3 ± 3.2		34.7 ± 15.1				
*Technique*							*F* (1, 2) = 14.316; *P*=0.001 (0.346)	*F* (1,2) = 0.632; *P*=0.539 (0.045)
Baseline	45.0 ± 3.7	6.71	44.7 ± 5.9	4.17	42.7 ± 7.4	11.91		
Posttest	47.9 ± 2.7		46.4 ± 5.2		46.4 ± 5.1				
DFOS total							*F* (1, 2) = 19.894; *P*=0.001 (0.424)	*F* (1,2) = 0.354; *P*=0.705 (0.026)
Baseline	81.1 ± 5.4	7.00	79.7 ± 10.8	5.21	75.8 ± 12.9	8.94		
Posttest	86.6 ± 4.2		83.2 ± 7.9		81.1 ± 8.4				
*DFOS-subjective*							*F* (1, 2) = 9.839; *P*=0.004 (0.276)	*F* (1,2) = 0.299; *P*=0.744 (0.022)
Baseline	81.5 ± 8.1	4.99	82.3 ± 7.7	5.91	78.5 ± 11.7	10.16		
Posttest	85.5 ± 10.1		86.8 ± 7.5		85.4 ± 9.6				

## Data Availability

The data used to support the findings of this study are available from the corresponding author upon request.
